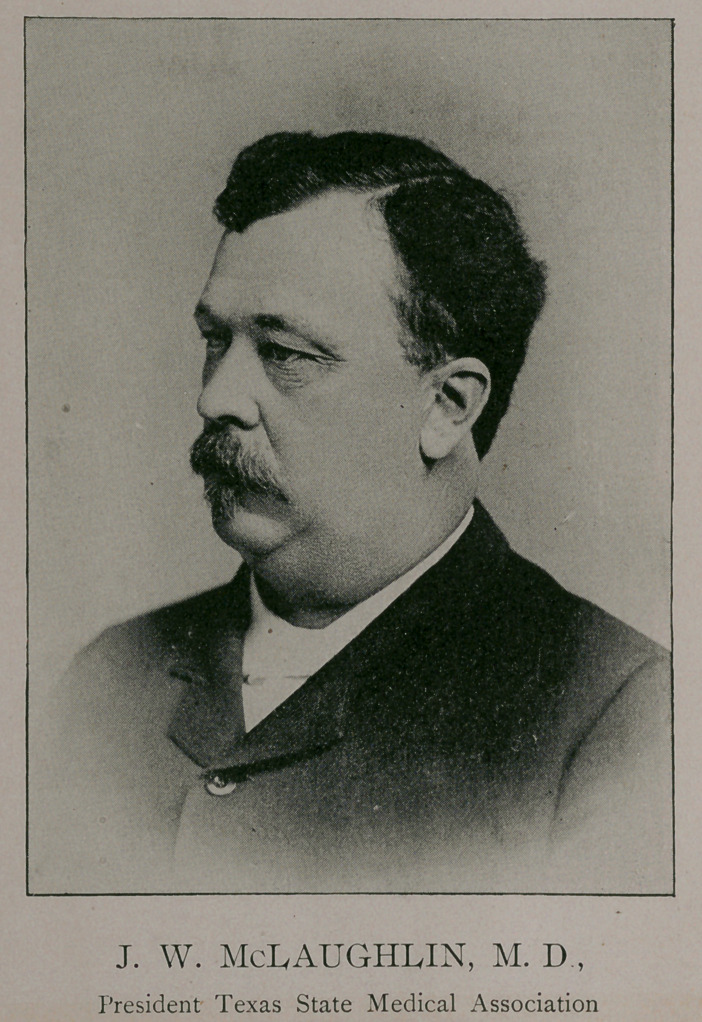# Biographical—President-elect J. W. McLaughlin, M. D.

**Published:** 1894-05

**Authors:** 


### PRESIDENT-ELECT J. W. McLAUGHLIN, M. D.

Dr. James Wharton McLaughlin, Austin, Texas, whose portrait the JOURNAL presents herewith, was born September 7th, 1840, at Springfield, Ohio. Father's name, Cyrus Duncan McLaughlin; mother's name, Sarah (Wharton) McLaughlin; paternal grandfather, James Wilson McLaughlin. He is of Scotch-English parentage.

Dr. McLaughlin was educated in the common schools of Ohio, and began the study of medicine at 16 years of age with his uncle, Dr. Andrew Campbell McLaughlin, of Tremont City, Ohio, and supported himself during the time. He attended his first course of medical lectures at Cincinnati College of Medicine and Surgery at Cincinnati, Ohio, during the session of 1859 and 1860; had but fifteen dollars at the beginning of the session, and managed to live on this amount until its close, besides paying $1.50 for shoes, and 10 cents on several occasions for entrance into the peanut gallery of the theatre.

At the close of the session he returned to Tremont City and entered into practice with his preceptor.

During the political excitement in the fall and winter of 1860 and spring of 1861, he was an active and outspoken advocate for States Rights, an uncompromising Democrat by inheritance and conviction, who regarded the Federal Union as a compact between the States, whereby the latter surrendered to the former certain rights designated in the Constitution, and reserved all other rights to themselves, and therefore believed, when the Southern States failed to obtain their rights within the Union, they should be allowed to withdraw from it peaceably. These views, as may be supposed, were not only very unpopular in that section, but marked their advocates as objects of public fury; consequently he left his native State between two suns, and in Louisville, Ky., in March, 1861, joined the 1st Kentucky Regiment of (Confederate) Infantry, was elected Lieutenant of Co. D of this regiment, at their reorganization, just before the first battle of Manassas, and served in this capacity until the regiment was disbanded, immediately after the peninsular campaign in Virginia. He then joined Gilmore's Company of Scouts of the 14th Virginia Cavalry, and afterwards served with Generals Morgan and Forrest, until the close of the war. Came to Texas in the fall of 1865, and immediately began a review of his former medical studies.

In January, 1866, he became associated with Dr. Sam D. McLeary, of Colorado county, Texas, in the practice of medicine. In the fall of the same year attended a second course of medical lectures at the Medical Department of the University of Louisiana, and graduated from this school in the spring of 1867; then located at Fayetteville, Fayette county, Texas, and was fortunate in securing, from the beginning, a lucrative practice. Married Miss Tabitha Bird Moore, the only child of Dr. Bird and Sarah E. Moore, of Fayette county, in September, 1867. They have six children—three boys and three girls—all living. Moved to Austin, Texas, in January, 1870, where he has since resided.

Dr. McLaughlin is a member of the following medical societies, viz.: Travis County Medical Society, Austin District Medical Society, Texas State Medical Association, American Medical Association, American Public Health Association, Pan-American Medical Congress and Southern Surgical and Gynecological Association.

He is the author of various medical papers which have appeared in Daniel's TEXAS MEDICAL JOURNAL, *N. O. Medical and Surgical Journal*, *Medical Record* and *American Therapist;* also, of a volume of 240 pages published in 1893, entitled "Fermentation, Infection and Immunity," which has received flattering reviews from home and foreign medical journals. Has been President of the Travis County Medical Society, the Austin District Medical Society, and, at the New Orleans meeting of the A. M. A., he was elected by the Texas delegates to represent the State in the organization of the Ninth International Medical Congress which was held in Washington, D. C.

Dr. McLaughlin is also a member of the "Texas Academy of Science" and the "Austin Microscopic Society."

In 1885, during the prevalence of the last epidemic of dengue in the State, he conducted original investigations into the etiology of this disease, and obtained from the blood of dengue sufferers in about forty different cases, and in all examined, a unique micrococcus which, he claims, is the specific cause of this disease. Artificial cultures of this micro-organism were made and its life-history carefully studied. From these it was found to possess characteristics in its method of grouping that differentiates it from all other known micrococci, and, therefore, establishes a relationship between this micro-organism and dengue.

A paper setting forth these original investigations, and the conclusions arrived at, was read by its author at the meeting of the A. M. A., held in St. Louis in 1886, and published in the Association journal.

## Figures and Tables

**Figure f1:**